# Acceptance of shared decision making with reference to an electronic library of decision aids (arriba-lib) and its association to decision making in patients: an evaluation study

**DOI:** 10.1186/1748-5908-6-70

**Published:** 2011-07-07

**Authors:** Oliver Hirsch, Heidemarie Keller, Tanja Krones, Norbert Donner-Banzhoff

**Affiliations:** 1 Department of General Practice/Family Medicine, University of Marburg, Marburg, Germany

## Abstract

**Background:**

Decision aids based on the philosophy of shared decision making are designed to help patients make informed choices among diagnostic or treatment options by delivering evidence-based information on options and outcomes. A patient decision aid can be regarded as a complex intervention because it consists of several presumably relevant components. Decision aids have rarely been field tested to assess patients' and physicians' attitudes towards them. It is also unclear what effect decision aids have on the adherence to chosen options.

**Methods:**

The electronic library of decision aids (arriba-lib) to be used within the clinical encounter has a modular structure and contains evidence-based decision aids for the following topics: cardiovascular prevention, atrial fibrillation, coronary heart disease, oral antidiabetics, conventional and intensified insulin therapy, and unipolar depression. We conducted an evaluation study in which 29 primary care physicians included 192 patients. After the consultation, patients filled in questionnaires and were interviewed via telephone two months later. We used generalised estimation equations to measure associations within patient variables and traditional crosstab analyses.

**Results:**

Patients were highly satisfied with arriba-lib and the process of shared decision making. Two-thirds of patients reached in the telephone interview wanted to be counselled again with arriba-lib. There was a high congruence between preferred and perceived decision making. Of those patients reached in the telephone interview, 80.7% said that they implemented the decision, independent of gender and education. Elderly patients were more likely to say that they implemented the decision.

**Conclusions:**

Shared decision making with our multi-modular electronic library of decision aids (arriba-lib) was accepted by a high number of patients. It has positive associations to general aspects of decision making in patients. It can be used for patient groups with a wide range of individual characteristics.

## Background

In shared decision making (SDM), patients are empowered in a way that they actively ask questions and participate in decisions about their health care on the basis of their preferences and values [1,2]. In clinical practice, patients are not regularly asked about their preferences [3]. Scheibler *et al*. [4] present results where about one-half of patients want to be involved in decision making, but a far smaller percentage of them are actually involved. The willingness of physicians to involve their patients in decision making is considerably lower than the preference of their patients. Physicians need to assess their patients' preferences before starting to discuss the reason for consultation [5].

Decision aids based on SDM are designed to help patients make informed choices among diagnostic or treatment options by delivering evidence-based information on options and outcomes. They are supposed to supplement the counselling process and can be delivered in different formats before, during, or after the consultation [6]. Decision aids are reported to increase knowledge, reduce decisional conflict, cause greater satisfaction with decision making, support more realistic expectations, achieve a greater likelihood of being able to make a decision, result in an increased association between patient values and decisions, support patient participation, and enhance communication between physicians, patients, and their relatives [7]. Decision aids should not substitute personal counselling because uncertain patients would then be abandoned [8]. Several authors argue for the need to develop evidence-based decision aids for a wide range of clinical applications that should display this evidence on a basic level to be understandable for the patient. They should be interactive so that individual risk data can be entered, and the effects of certain treatments can be seen immediately. Pros and cons can be discussed by using weighted scales [1].

A patient decision aid can be regarded as a complex intervention because it consists of several presumably relevant components. Therefore, there is a need to model components of a complex intervention and to perform exploratory trials to pre-test preliminary versions of an intervention. Outcomes of potential relevance like patient characteristics might then represent endpoints in later controlled trials [9]. Consequently, decision aids require rigorous field testing to assess patients' and physicians' attitudes towards them; recently, several studies attempted to carry out such field tests [10-12]. Therefore, it is strongly recommended to evaluate decision support systems in a real world setting with multi-perspective, multi-method studies ahead of dissemination for routine use. Such studies should contain a variety of aspects, use multiple methods, apply flexible study designs with longitudinal measures, and do formative and summative evaluations. Most studies in this area concentrate on physicians, not on patients or other users [1,11,13-15].

We undertook a mixed method evaluation study using quantitative and qualitative methods (patient interviews, focus groups). The importance of mixed method research in complex interventions like decision aids is underlined by the study of Protheroe *et al*. [9]. In their pragmatic randomized controlled trial of a decision aid for women who attended their primary care physician because of menorrhagia, they found that women with less formal education reported greater benefits in qualitative measures. In contrast, the quantitative analysis revealed that women with more formal education benefited most from the intervention. This inter-method discrepancy emphasizes the need for a multi method approach when examining such a complex intervention.

The aim of our study was to evaluate the acceptance of SDM with reference to an interactive, transactional, and evidence-based library of decision aids by patients and physicians in the primary care context.

## Methods

We performed a mixed method evaluation study. According to the taxonomy of mixed methods as designed by Palinkas *et al*. [16], we sequentially collected quantitative and qualitative data. Our intention was to use the qualitative method to answer questions raised by quantitative data (function: expansion). We intended to build the qualitative data upon our quantitative data set (process: connect). Here, we present quantitative data of patients on the acceptance of SDM with reference to our electronic library of decision aids (arriba-lib) and its association with decision making. The analyses of our comprehensive qualitative data and the integration of quantitative and qualitative data will be presented in different publications.

### arriba-lib

**Figure 1:**
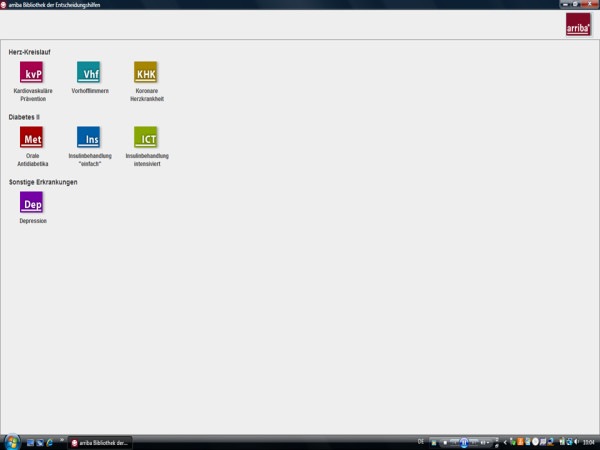
**Opening screen of arriba-lib**.

Our electronic library of decision aids, arriba-lib, is an extension of ARRIBA-Herz, a decision aid on cardiovascular prevention that was investigated in a randomised controlled trial [17], and which is now named 'arriba™.' The software, whereby 'lib' is an acronym for 'library,' has a modular structure and presently contains evidence-based decision aids for the following topics: cardiovascular prevention, atrial fibrillation, coronary heart disease, oral antidiabetics, conventional and intensified insulin therapy, and unipolar depression. Further modules are currently in development. Figure 1 displays the opening screen of arriba-lib and shows the library-like structure. It is a Java application that does not need an installation process and is less than 15 megabyte.

**Figure 2:**
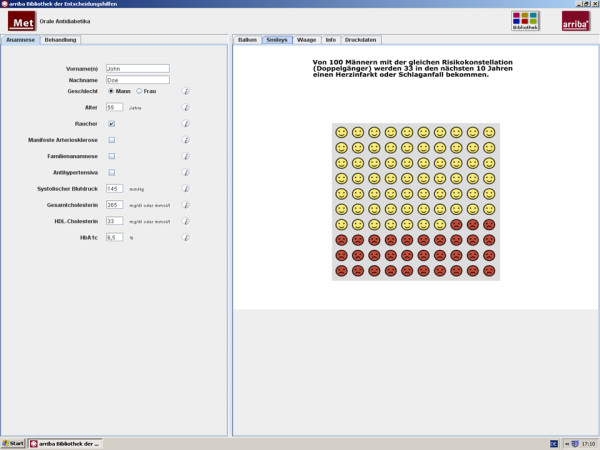
**Individual risk information with smileys**. Within the module regarding oral antidiabetics (metformin), the risk to suffer from a myocardial infarction or stroke in the next ten years compared to one hundred patients with the same characteristics is shown examplarily with smileys.

**Figure 3:**
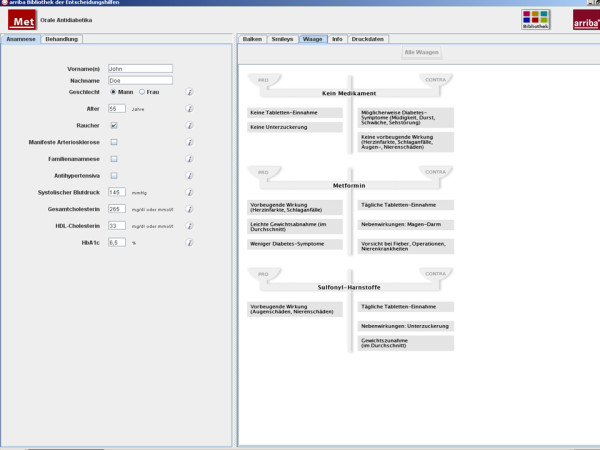
**Weighted scales in arriba-lib for the deliberation phase**.

The modules are structured to assist physicians in counselling their patients according to the philosophy of SDM [18,19]. In our programme, this process comprises the following successive steps: definition of the problem, discussion of the individual risk, discussion of treatment options, deliberation, and plan for future actions where 'no treatment' is also a possible choice. These steps can be regarded as a framework to help the clinician to effectively structure the encounter. After typing in history information, individual risk information is displayed by smileys, bar charts, or curves (Figure 2). These smileys are an easy-to-understand graphic representation of risk information that takes the limited numeracy and statistical literacy of patients and physicians into account [20]. The presentation of global risk information was shown to increase the accuracy of perceived risk [21]. Risk-reducing effects can be demonstrated after choosing between evidence-based treatment options. The process of deliberation can be supported by weighted scales mentioning pros and cons related to each option (Figure 3).

Additional evidence-based information on clinical topics and communication strategies is also provided in the programme and can be easily accessed in each module. For the purpose of our study, log files of every consultation were created that recorded every step taken in the modules and how long it took to initiate the next step. The results of these analyses will be presented separately.

The participating physicians received a personal introduction into the programme and the philosophy of SDM by seminars, outreach visits, and a brochure explaining details of the programme.

### Recruitment and sampling

**Figure 4:**
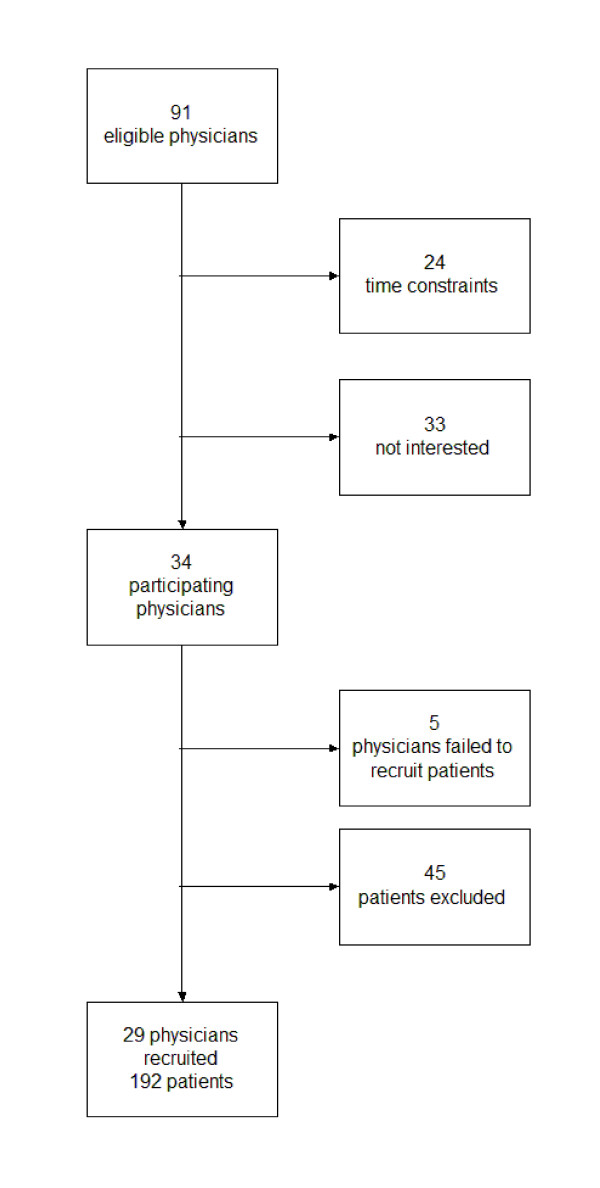
**Flow chart displaying the recruitment process in the arriba-lib study**.

We invited a convenience sample of 91 primary care physicians in the German federal lands of North Rhine-Westphalia and Hesse to participate in our study, of which 34 agreed. Five of these 34 physicians failed to recruit any patients, leaving 29 participating primary care physicians who included 192 patients. Patients were included when there was a decision to be made in the topics covered by arriba-lib regardless of the stage of the underlying disease. Physicians were told that they should stop recruitment when they had included 10 consecutive patients. Twenty-seven patients refused to participate, and 18 patients fulfilled our exclusion criteria (restrictions because of language, cognitive abilities, psychiatric disorder, and severity of somatic disease). On average, recruitment of patients comprised a period of eight weeks. The recruitment process is depicted in Figure 4.

The study complies with the Declaration of Helsinki. The research protocol was approved by the local research ethics committee at the University of Marburg. All physicians and patients gave their written informed consent. Our study corresponds to Phase II of the model for complex interventions by the British Medical Research Council [22].

### Measurements

After each consultation, the physician and the patient filled in questionnaires. The patient questionnaire consisted of the SDM Questionnaire (SDM-Q), which evaluates nine theoretical stages of the SDM process according to theoretical frameworks [23], and the Patient Participation Scale (PPS) [24] to measure patient satisfaction and participation that consists of six items which can be rated as follows: totally agree, agree, neither...nor, disagree, or totally disagree. High scores signify low participation in, as well as low satisfaction with, the consultation. Additional details on these questionnaires are presented in Hirsch *et al*. [25]. We further included questions on who made the decision, who should make the specific treatment decision, and a global rating of satisfaction with the encounter.

Qualitative semi-structured patient interviews were conducted within one week after the consultation with those patients who had agreed to be personally interviewed. In those interviews, questions were asked about the acceptance of arriba-lib. After 20 interviews, saturation was reached. Analyses of these qualitative data will be published separately. Two months after the consultation, patients were asked in structured telephone interviews whether a decision had been made after the consultation with arriba-lib, whether the decision had been implemented, and whether they would like to be counselled again with arriba-lib.

### Statistical methods

Because of the hierarchical structure of our data (patients nested within physicians), we used generalised estimation equations (GEE) to measure associations within patient variables [26]. The Wald χ^2^-test was used as a test statistic. To enhance the interpretability of the results, we also analysed the data with traditional crosstab analyses (χ^2^-test, Haldane-Dawson test, contingency coefficient). Effect sizes Cramer V and Cohen's *d *were used to measure the meaning of associations and differences [27]. Because of the exploratory nature of our study, we decided not to adjust for multiple testing. This has to be considered when interpreting the results [28].

After inspection of descriptive data, there was a maximum of 10% missing data on isolated variables that we assumed as missing completely at random because there were no patterns of associations with other variables [29]. Imputation of missing data was performed by inserting the means of the respective variables on physician level; in simulation studies, this was found to be most appropriate when the data had a hierarchical structure [30].

## Results

The average age of the 29 participating primary care physicians was 52.2 years (sd 5.1 years; range: 43 to 64 years). Eighteen were male (62%). The average time practicing was 14 years (sd 7.5 years; range: 0 to 27 years).

The module for cardiovascular prevention was selected in 128 patients (67%), the diabetes modules in 43 patients (22%), coronary heart disease in 8 patients (4%), atrial fibrillation in 8 patients (4%), and depression in 3 patients (2%).

The 45 patients who had been excluded from the study did not differ significantly from participants regarding gender (χ^2 ^= 1.69, *p = *0.19) and age (t = 1.69, *p = *0.09; *d *= 0.28). The medium age of the 192 participating patients was 62.4 years (sd 11.8 years; range: 23 to 83 years). There was an equal distribution regarding gender, with 97 males (50.5%) and 95 females (49.5%). A majority of 70.3% had a formal education of eight years or less, 14.6% had a formal education of up to 10 years, and 15.1% of more than 10 years. In our sample, 122 patients (63.5%) preferred SDM with their physicians, and exactly the same proportion mentioned that SDM actually had taken place.

In 46 patients (24.0%) the reason for consultation was a check-up, 34 patients (17.7%) attended their physicians for a monitoring visit, and 20 patients (10.4%) were seen in the context of a disease management programme. The remaining patients came with acute complaints or to discuss results of laboratory examinations.

Two months after the consultation 133 patients (69.3% of the original sample) took part in a short telephone follow-up interview.

### Acceptance of and satisfaction with shared decision making and arriba-lib in patients

Table 1 depicts the patient's data on the items of the SDM-Q nested under different steps of the SDM process. There were high ceiling effects, and a floor effect in step five, where only a small fraction of patients had mentioned other possibilities that their doctor had not referred to. The majority of patients perceived that different aspects of the SDM process had actually taken place.

**Table 1:** Steps of the shared decision making process as reported by patients in the Shared Decision Making Questionnaire (SDM-Q).

SDM-Q	'agree'(%)
**Step 1: Disclosure that a decision needs to be made**	
My doctor told me that a treatment decision is necessary.	**83.3**

**Step 2: Formulation of equality of partners**	
My doctor asked me if I want to participate in decision making.	**91.7**

**Step 3: Equipoise statement**	
Due to my medical condition, a treatment decision based on the physicians' recommendation is already clear.	**78.6**

**Step 4: Informing on the options' benefits and risks**	
My doctor has informed me about a variety of alternatives.	**85.9**
The possibility to choose no treatment was also discussed.	**72.9**

**Step 5: Investigation of patient's understanding and expectations**	
I have mentioned other possibilities that my doctor has not referred to.	**19.8**
My doctor has asked me what I think about different treatment options.	**77.6**

**Step 6: Identification of preferences (both)**	
I have communicated to my doctor which decision I prefer.	**74.5**
My doctor has told me which decision he prefers.	**90.6**

**Step 7: Negotiation**	
In the selection of a treatment method, my thoughts were taken into account just as much as the considerations of my doctor.	**97.4**
My doctor and I thoroughly considered the different treatment options.	**93.2**

**Step 8: Shared decision making**	
My doctor enabled me to actively participate in decision making about treatment.	**88.5**
My doctor and I selected a treatment together.	**88.5**

**Step 9: Arrangement of follow-up**	
My doctor and I reached an agreement as to how we will proceed.	**92.2**

Table 2 lists means and standard deviations of the items of the Patient Participation Scale (PPS). The means of all the items of the PPS were between the rating categories 'totally agree' and 'agree.' Therefore, patients were highly satisfied with the encounter. In a global rating, a very high proportion of patients (97.4%) were 'very satisfied' or 'satisfied' with the consultation.

**Table 2:** Means and standard deviations of patient ratings on the Patient Participation Scale.

	Mean (sd)
1. My doctor helped me to understand all of the information.	**1.18 (0.39)**

2. My doctor understood what is important for me.	**1.18 (0.40)**

3. My doctor answered all of my questions.	**1.17 (0.47)**

4. I was sufficiently involved in decisions about my treatment.	**1.22 (0.48)**

5. I have decided the further treatment together with my doctor and I am satisfied with the result.	**1.33 (0.68)**

6. I am satisfied with the manner by which my treatment has been discussed and decided.	**1.20 (0.47)**

The scale ranges from 1 (totally agree) to 5 (totally disagree).

Two months after the consultation, patients were asked in a telephone interview whether they would like to be counselled again with arriba-lib. Of those asked, 65.2% wanted to be counselled again with arriba-lib, 18.9% had no defined preference, and 15.9% did not remember the decision aid. There was no significant association between gender and the further preference for arriba-lib in patients (GEE: Wald-χ^2 ^= 1.35, df = 1, *p = *0.25). Furthermore, there were no significant associations between age and preference for arriba-lib (GEE: Wald-χ^2 ^= 0.20, df = 1, *p = *0.65) and between education and further preference for arriba-lib (GEE: Wald-χ^2 ^= 6.11, df = 4, *p = *0.19).

### Association of arriba-lib with decision making and exploration of additional factors

In 70% of the responding patients, we found a perfect match between preferred and perceived decision making. Fifty-seven percent of the patients had preferred and actually perceived SDM. This resulted in a contingency coefficient of 0.65 (*p *< 0.001) and a high effect size (Cramer V = 0.39).

Of those patients reached in the telephone interview, 69.9% said that a decision had been made, and 80.7% of them had implemented the decision. We found no significant associations between gender (GEE: Wald-χ^2 ^= 1.62, df = 1, *p = *0.20) or education (GEE: Wald-χ^2 ^= 1.55, df = 4, *p = *0.82) and the patient's indication that a decision had been made. Elderly patients were more likely to say that no decision could be made (GEE: Wald-χ^2 ^= 7.76, df = 1, *p = *0.005).

The implementation of patients' decisions was independent from gender (GEE: Wald-χ^2 ^= 0.37, df = 1, *p = *0.54) and education (Haldane-Dawson-Test: z = 0.24, *p = *0.82). Elderly patients were more likely to say that they implemented the decision (GEE: Wald-χ^2 ^= 4.58, df = 1, *p = *0.03).

## Discussion

We conducted a study to evaluate the acceptance of SDM with reference to an interactive, transactional, and evidence-based library of decision aids and its associations with decision making in patients in primary care practice. The majority of patients perceived that different aspects of the SDM process had actually taken place. Patients were highly satisfied with the encounter. In a brief telephone interview two months after the consultation, two-thirds of the patients stated that they would like to be counselled again with arriba-lib.

There was a high match between preferred and perceived decision making in patients. More than two-thirds of patients said that a decision could be made after the consultation. This was not associated with gender or education, but elderly patients were more likely to say that no decision could be made. More than three-quarters of those reached in the telephone interview implemented the decision within an interval of two months after the consultation. Elderly patients were more likely to say that they had implemented the decision.

Our study has several limitations. It is possible that physicians did not necessarily perform consecutive patient recruitment, and instead treated some patients as usual. This may have led to a positive selection of patients who were already favourably inclined to SDM. This positive selection bias concerning SDM may also be true of the participating physicians because just 32% of the invited physicians took part in our study. Results of statistical analyses within a small evaluation study should always be treated with caution and should be regarded as preliminary [28]. We had no control group in our study, so we cannot compare our results to the situation of usual care.

There is a lack of an accepted primary outcome regarding the use of decision aids. Possible categories to classify measures of effectiveness are knowledge, decision process (*e.g*., satisfaction and participation preference), decision outcomes (*e.g*., has a treatment decision been made, adherence), health status, and economic measures. In our sample, patients were highly satisfied with arriba-lib and the process of SDM. Whether this can be solely attributed to the programme is debatable. In a different study, we found that patients were highly satisfied with their physicians regardless of SDM being applied or not [25]. This challenges patient satisfaction to be an adequate measure in evaluating decision aids. It further has to be mentioned that the questionnaires used may have primarily measured satisfaction with the SDM process or just patient satisfaction with their physician in general. Our telephone interviews have the same limitations than other telephone surveys, *e.g*., social desirability. Validity checks were not possible because we were not allowed to view patient records. It was not recorded what kind of decisions had been made. We were primarily interested in the acceptance of an SDM approach in connection with our electronic library. The implementation of a decision also depends on what kind of decision was made.

Congruence between preferred and perceived decision making predicted adherence to medical decisions, while age and gender did not have any explanatory power [31]. Our results partially support these conclusions of Jahng *et al*. because we also found a high congruence between preferred and perceived decision making and a high adherence to decisions.

Our findings suggest that our electronic library of decision aids has a positive association with decision making in patients and that future preference for it is high, regardless of patient characteristics like gender, education, or age. Sepucha *et al*. [32] report that people with lower education, lower income, and high trust in their doctor overestimate their state of being informed about medical issues. Therefore, it is important that physicians check the level of understanding in patients during consultation with a decision aid.

In their updated systematic review, Légaré *et al*. found time constraints, patient characteristics, and the clinical situation to be the most often reported barriers for the implementation of SDM [33,34]. On the basis of our results, we agree with the authors that physicians should not assume that patients with certain sociodemographic characteristics are not fit for SDM. Instead, the encounter should be adapted to the individual patient.

Consequently, there is a need for constant evaluation of measures used in the area of SDM. In the near future, we will conduct a randomised controlled trial that will attempt to find active ingredients in the risk-presenting part of the programme. Different methods of risk presentation will be applied. Physicians and patients will be asked which presentation they find most suitable. In another study, we will check the validity of our cardiovascular prevention module by longitudinal epidemiological data.

## Conclusions

SDM with reference to our comprehensive electronic library of decision aids (arriba-lib) was accepted by a high number of patients. It has positive associations with general aspects of decision making in patients and can be used for patient groups with a wide range of individual characteristics.

## Competing interests

The authors declare that they have no competing interests.

## Authors' contributions

OH participated in the study design and coordination, developed the concept for data analysis, carried out the study, performed the statistical analyses, and drafted the manuscript. HK participated in the study design and coordination, the rationale for the data analyses, carried out the study, and helped to draft the manuscript. TK participated in the study design and coordination, the rationale for the data analyses and helped to draft the manuscript. NDB participated in the study design and coordination, the rationale for the data analyses, and helped to draft the manuscript. All authors read and approved the final manuscript.
